# The moderating role of the case-mix index in the relationship between medical staff allocation and average cost per inpatient visit

**DOI:** 10.3389/fpubh.2026.1791368

**Published:** 2026-03-19

**Authors:** Change Xiong, Ying Xia, Shandan Xu, Jing Xiao

**Affiliations:** 1 School of Public Health, Wuhan University of Science and Technology, Wuhan, China; 2 Department of Human Resources and Science Education, Hubei Provincial Center for Disease Control and Prevention, Wuhan, China

**Keywords:** case-mix index, health resource, medical cost, medical staff allocation, moderate effect

## Abstract

**Background:**

Greater medical staff allocation and higher Case-Mix Index (CMI) values are both associated with increased patient medical expenditures. However, it remains unclear whether CMI moderates the relationship between medical staff allocation and medical expenditures. The present study focus on whether medical staff allocation predicts the cost per inpatient visit, whether this association is enhanced under conditions of high CMI.

**Aim:**

To explore the mechanism underlying the role of the CMI in the relationship between medical staff allocation and the average cost per inpatient visit.

**Methods:**

Data were collected from 207 general hospitals in Hubei Province in 2019 using the cluster sampling method. Pearson’s correlations were used to examine the associations between medical staff allocation, the CMI and the cost per inpatient visit. The moderating role of the CMI between medical staff allocation and the average cost per inpatient visit was analyzed using Model 1 of the PROCESS macro.

**Results:**

The number of patients per doctor (*r* = −0.180, *p* < 0.01), beds per nurse (*r* = −0.181, *p* < 0.01), beds per doctor (*r* = −0.225, *p* < 0.01) and the average cost per inpatient visit showed a significant negative correlation. The CMI positively moderated the relationship between the average cost per inpatient visit and both the number of beds per doctor (*p* < 0.01) and the number of beds per nurse (*p* < 0.01). The moderating effect was stronger when hospitals had high CMI value influencing average cost per inpatient visit.

**Conclusion:**

CMI value can enhance the negative effects of the number of beds per doctor or nurse on average cost per inpatient visit, revealing that rational CMI benchmark for the hospital or prioritizing disease categories with clinically appropriate treatment complexity can mitigate the extent to which healthcare workforce allocation influences rising medical expenditures. Reasonable medical staff allocation and the CMI serve as important factors in controlling the average cost per inpatient visit.

## 1 Introduction

The average cost per inpatient visit is not only a direct reflection of how expensive it is for local people to visit a doctor but is also an important indicator of the level of regional medical efficiency. Since the implementation of the new round of medical and health system reforms in 2009, resulting in full coverage of the basic medical insurance system and the gradual expansion of public hospital reform, increases in the average cost per inpatient visit in China have been somewhat controlled. According to data from the China Health Statistical Yearbook, the average cost per inpatient visit in public hospitals has increased from 6,416 yuan in 2010 to 11,469 yuan in 2022, a 0.79-fold increase. However, the problem of unreasonable growth in medical costs still exists.

In order to control the unreasonable growth of medical costs, China began to implement the Disease Diagnosis Related Groups (DRGs) payment system in 2019, with 30 cities as national pilot cities for DRGs payment. In July 2024, DRGs payment was implemented nationwide. The case mix index (CMI), as an important index of the DRG system, reflects the average comprehensive medical capacity of medical institutions and the average severity or difficulty of diseases (1). CMI is an objective metric calculated by summing the Medicare Severity-Diagnosis Related Group weights for each encounter and dividing by the number of encounters (2). Its research (3) mainly focuses on hospital resource allocation and medical performance management, and it is more in line with the actual work situation by adjusting the hospital assessment indexes to evaluate the work efficiency through the CMI value. The higher the CMI is, the higher the risk and complexity of the disease, and the more care workload in the department.

Studies of the relationship between the CMI and healthcare costs indicate that the higher the CMI value, the higher the healthcare costs. Li et al. (4) found that with a significant increase in the CMI, patients with more severe conditions, in terms of physical and nutritional status, would spend more on healthcare costs. A survey in Greek public hospitals (5) indicated that an increased severity of illness and increased complexity of certain cases may contribute to a higher average cost per inpatient visit. A study by Schlosser et al. (6) showed that the higher the CMI, the higher the annual cost per case incurred in wards with a higher CMI compared to conventional wards. In contrast, Syed et al. (7) investigated patients undergoing surgery for urologic tumors in both academic and community hospitals and found that, despite the similarity in the CMI between the two hospitals, patients in the academic hospitals had a significantly higher average cost per inpatient visit. Collectively, the aforementioned studies demonstrate a positive association between the Case Mix Index (CMI) and healthcare expenditures. These findings collectively support the theoretical foundation underlying the present study’s hypothesis.

The CMI, as an indicator of the intensity and complexity of resource consumption in hospitals, is often influenced by medical staff allocation. Medical staff allocation is usually reflected by the ratio of healthcare staff (mainly doctors and nurses) to beds or patients, that is, the “number of beds per nurse”, “number of beds per doctor”, “number of patients per nurse” and “number of patients per doctor” (8, 9). In China, bed is frequently adopted as the primary criterion for allocating health human resources, whereas international practice typically uses patient volume—such as annual inpatient admissions or outpatient visits—as the foundational metric for such allocation. Given that this study seeks to identify four key indicators to assess core human resource allocation in hospitals, the choice of benchmark must align with internationally recognized standards of health system performance and equity. When doctors are understaffed, hospitals may tend to admit patients with minor illnesses to maintain operational efficiency, resulting in an artificially low CMI; conversely, adequate doctors (especially senior doctors) enhances the ability to deal with difficult and serious illnesses, proactively attracting or taking on more complex cases, which pushes up the CMI. In addition, nurse staffing can influence CMI. Yang et al. (10) reported that the CMI was positively correlated with the nursing workload, as reflected by the number of beds per nurse in the ward. Medical staff allocation has a direct impact on a hospital’s ability to handle cases. For hospitals with a higher CMI, staffing of more experienced physicians and nurses can assist with the more efficient handling of complex cases, thus potentially mitigating increases in the average cost per inpatient visit. The existing literature on CMI and the allocation of health workforce informed the theoretical underpinnings of this study’s hypotheses.

At present, the relationship between healthcare staffing and healthcare costs is unclear. Previous studies of large groups of medical-surgical inpatients (11) have shown that lowering the patient-to-nurse staffing ratio not only meets the hospital’s requirements for ensuring a CMI consistent with the hospital’s strategic priorities but also results in better patient outcomes, which in turn reduces hospitalization costs. However, Ross et al. (12) investigated lung cancer patients undergoing lobectomy and found that, in the presence of a lower number of patients per nurse, the average cost per inpatient visit was increased. There are many other published studies investigating medical staff allocation levels and the average cost per inpatient visit. For example, a study of inpatients at one hospital (13) showed that a decrease in the patient-to-nurse staffing ratio from 1:6 to 1:3–4 was one of the reasons why the average cost per inpatient visit for patients with COVID-19 was much higher than that for patients without COVID-19. Herrin et al. (14) found that increasing the number of nurses per bed led to increased nursing costs for hospitalization. Drawing on the existing literature, we formulate a hypothesis concerning the association between the ratio of medical staff allocation and medical expenditure. The specific hypotheses are detailed below.

*H*1a: The number of beds per doctor (the number of beds each physician manages) will be negatively associated with the average cost per inpatient visit.

*H*1b: The number of beds per nurse (the number of beds each nurse manages) will be negatively associated with the average cost per inpatient visit.

*H*1c: The number of patients per doctor (how many patients each physician cares for) will be negatively associated with the average cost per inpatient visit.

*H*1d: The number of patients per nurse (how many patients each nurse cares for) will be negatively associated with the average cost per inpatient visit.

**Figure 1:**
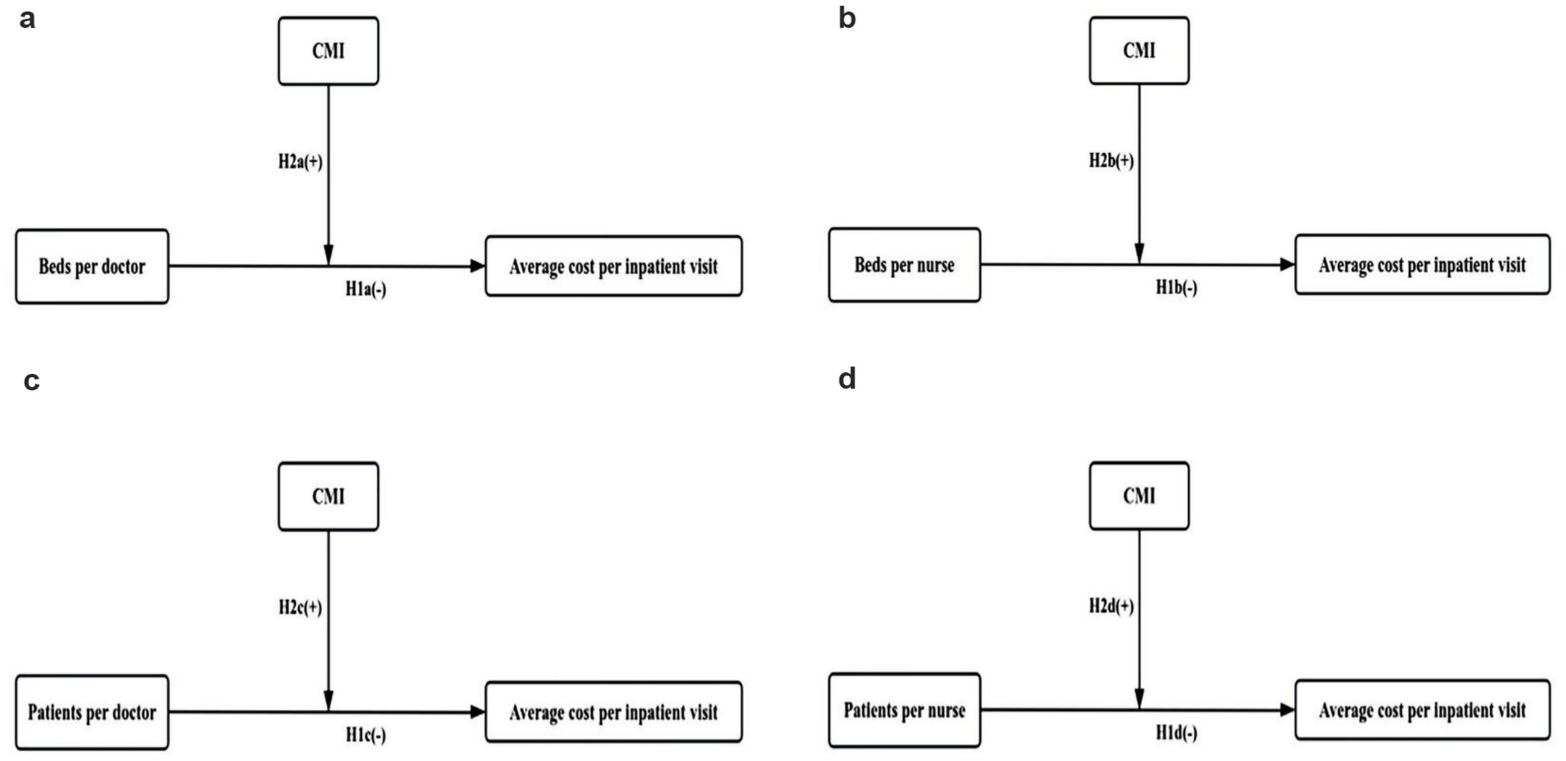
Hypothetical model. **(a)** CMI will moderate the relationships between the number of beds per doctor and the average cost per inpatient visit. **(b)** CMI will moderate the relationships between the number of beds per nurse and the average cost per inpatient visit. **(c)** CMI will moderate the relationships between the number of patients per doctor and the average cost per inpatient visit. **(d)** CMI will moderate the relationships between the number of patients per nurse and the average cost per inpatient visit.

Given the above inconsistency in the published literature, there is a need to further study the relationship between medical staff allocation and healthcare costs. Further, in the context of the implementation of the DRG payment system nationwide, the role of the CMI in the relationship between medical staff allocation and the average cost per inpatient visit in China is worthy of exploration. Based on the above literature, it was hypothesized that a lower ratio of medical staff per bed would be associated with a higher average cost per inpatient visit. Further, it was predicted that the CMI would positively moderate this relationship. To test this hypothesis, medical staff allocation was measured using four indicators: the number of beds per doctor (BDR), the number of beds per nurse (BNR), the number of patients per doctor (PDR) and the number of patients per nurse (PNR). The number of beds per nurse was equal to the number of actual beds divided by the number of practising (assistant) physicians. The number of beds per nurse was the number of actual beds divided by the number of registered nurses. The number of patients per doctor was the number of discharged patients divided by the number of practising (assistant) physicians. The number of patients per nurse was the number of discharged patients divided by the number of registered nurses. The specific hypotheses are detailed below (Figure 1).

*H*2a: The CMI will moderate the relationship between the number of beds per doctor and the average cost per inpatient visit.

*H*2b: The CMI will moderate the relationship between the number of beds per nurse and the average cost per inpatient visit.

*H*2c: The CMI will moderate the relationship between the number of patients per doctor and the average cost per inpatient visit.

*H*2d: The CMI will moderate the relationship between the number of patients per nurse and the average cost per inpatient visit.

This study employs a two-stage analytical framework: first, it examines bivariate correlations among all key variables; second, it conducts a series of multiple regression analyses to assess the moderating role of the interaction term between medical staff allocation and Case-Mix Index (CMI), with medical expenses as the dependent variable. Separate regressions are estimated for medical staff allocation, CMI, and their interaction term controlling for relevant covariates to rigorously evaluate moderation effects.

## 2 Methods

### 2.1 Study design

This study was a cross-sectional survey.

### 2.2 Sample and participants

The cluster sample was recruited from Hubei Province. Hubei Province is located in the middle of China and has abundant medical care resources. In 2019, there were 561 general hospitals, including 239 secondary and above general hospitals, 126 traditional Chinese hospitals and 311 specialty hospitals in Hubei Province. Hubei Province had 155,000 physicians, 196,000 registered nurses and 291,000 hospital beds in 2019. A total of 9,935,000 patients were discharged in 2019. All 239 secondary and above general hospitals in Hubei Province in 2019 were invited to participate in this study. Institutional questionnaires were used to obtain data from each hospital. Staff in the information department of each hospital were responsible for completing the questionnaire. A total of 207 valid institutional questionnaires were returned for analysis following the exclusion of invalid questionnaires and hospitals with substantial missing data. Traditional Chinese medicine hospitals, maternal and child health centers, specialty hospitals, township health centers and community service centers were excluded.

### 2.3 Measures

#### 2.3.1 Dependent variable

Our study uses average cost per inpatient visit as dependent variable.

#### 2.3.2 Independent variable

Our explanatory variable is medical staff allocation, including beds per doctor (BDR), beds per nurse (BNR), patients per doctor (PDR), patients per nurse (PNR).

Moderator variable is case-mix index (CMI). The CMI value is calculated using a standardized methodology, as specified in the following equation:


$$\mathrm{CMI}=\Sigma \;(\text{Weighti}\times Ni)/N_{t},$$

where Weightᵢ denotes the relative weight of DRG group i, Nᵢ represents the number of cases assigned to DRG group i, and Nt is the total number of cases.

#### 2.3.3 Control variables

Hospital characteristics will serve as the primary control variables in this study, including hospital grade, hospital affiliation, geographical distribution of hospital, medical income, medical income, bed utilization rate, and average length of hospitalization.

### 2.4 Statistical analysis

Descriptive statistics and Pearson correlation analysis were performed using SPSS 26.0. Model 1 of the PROCESS macro (15) was chosen to analyze the moderating effect of the CMI on the relationship between medical staff allocation and the average cost per inpatient visit, where the variables were standardized. In the case of a significant moderating effect, simple slope analysis was performed to test the difference in the effect of medical staff allocation on the average cost per inpatient visit at different levels of the CMI.

## 3 Results

### 3.1 Descriptive analysis

Of the 207 hospitals included in this study, the majority were secondary (61.3%) and public (88.9%) hospitals. Overall, the average healthcare revenue was (510.82 ± 117.57) million and the total visits was at (522580.60 ± 842515.40). 46 (22.2%) hospitals reported healthcare revenues greater than 500 million and 61 (29.5%) hospitals reported more than 500,000 visits. Further, 137 (66.2%) hospitals had a bed occupancy rate of 85% or more, while about half of the hospitals had an average length of stay of either more or less than 9 days. Geographically, there were 108 (52.2%) municipal hospitals, and the ratio of hospitals at the county-level and below/municipal hospitals was 91.6%. The mean CMI in the 207 hospitals was 0.89 (SD = 0.21), the mean number of patients per nurse was 145.33 (SD = 153.58), the mean number of patients per doctor was 118.89 (SD = 57.18), the mean number of beds per nurse was 2.39 (SD = 1.93), the mean number of beds per doctor was 3.88 (SD = 1.88) and the mean cost per inpatient visit was ¥7,655.65 (SD = 3956.81; Table 1).

**Table 1:** Hospital characteristics (*N* = 207).

Variable	N(%)
Grade	
Secondary hospital	127(61.3)
Tertiary hospital	80(38.7)
Hospital affiliation	
Private hospital	23(11.1)
Public hospital	184(88.9)
Geographical distribution of hospital	
Municipal hospital	108(52.2)
Hospital at the county-level and below	99(47.8)
Medical income (million)	
≤500	161(77.8)
>500	46(22.2)
Total visits	
≤500,000	146(70.5)
>500,000	61(29.5)
Bed utilization rate	
≤85	70(33.8)
>85	137(66.2)
Average length of hospitalization (days)	
≤9	107(51.7)
>9	100(48.3)

### 3.2 Pearson correlation analysis

Correlation analysis revealed significant correlations between the CMI, medical staff allocation, and the average cost per inpatient visit. The strongest correlation with the number of beds per doctor was the number of beds per nurse (*r* = 0.560, *p* < 0.01). The strongest correlation with the average cost per inpatient visit was the medical income (*r* = 0.691, *p* < 0.01). There was also a significant correlation between the CMI and the number of beds per nurse (*r* = −0.259, *p* < 0.01) and beds per doctors (*r* = −0.343, *p* < 0.01; Table 2).

**Table 2:** Correlations among the study variables.

Variables	Average cost per inpatient visit	Hospital grade	Hospital affiliation	Geographical distribution of hospital	Medical income	Total visits	Bed utilization rate	Average length of hospitalization	CMI	Patients per nurse	Patients per doctor	Beds per nurse	Beds per doctor
Average cost per inpatient visit	1												
Hospital grade	0.577**	1											
Hospital affiliation	0.070	0.123	1										
Geographical distribution of hospital	0.135	0.124	−0.031	1									
Medical income	0.691**	0.369**	0.121	0.156*	1								
Total visits	0.687**	0.441**	0.167*	0.149*	0.972**	1							
Bed utilization rate	0.339**	0.372**	0.376**	0.017	0.340**	0.388**	1						
Average length of hospitalization	0.304**	−0.044	0.006	0.058	−0.054	−0.088	0.022	1					
CMI	0.631**	0.518**	0.195**	0.163*	0.520**	0.566**	0.463**	0.167*	1				
Patients per nurse	−0.059	0.008	0.149*	−0.111	−0.003	0.027	0.124	−0.130	0.016	1			
Patients per doctor	−0.180**	−0.009	0.358**	−0.127	0.090	0.117	0.470**	−0.282**	0.064	0.080	1		
Beds per nurse	−0.181**	−0.198**	−0.0798	−0.021	−0.128	−0.161*	−0.406**	0.0109	−0.259**	−0.044	−0.130	1	
Beds per doctor	−0.225**	−0.306**	−0.136	−0.133	−0.171*	−0.219**	−0.296**	0.179**	−0.343**	−0.129	0.286**	0.560**	1

**p < 0.01, *p < 0.05.

### 3.3 Analysis of moderating effects

As shown in Table 3, the direct effects of the number of beds per doctor (B = −0.28, *p* < 0.01, 95% CI [−0.35, −0.21]), number of beds per nurse (B = −0.22, *p* < 0.01, 95% CI [−0.34, −0.10]) and number of patients per doctor (B = −0.21, *p* < 0.01, 95% CI [−0.33, −0.85]) on the average cost per inpatient visit were all statistically significant. Therefore, hypotheses 1a, 1b and 1c were supported. The moderating effect of the CMI was significant in Model 1 (B = −0.14, *p* < 0.01, 95% CI [−0.57, 0.29]), Model 2 (B = −0.40, *p* < 0.01, 95% CI [−0.92, 0.12]). Therefore, hypotheses 2a and 2b were supported.

**Table 3:** Moderation analyses.

Model	Variables	B	SE	t	*p*	LLCI	ULCI	R^2^	ΔR^2^	F	*p*	Hypotheses is supported or not
Model 1	constant	−0.14	0.22	−0.65	0.52	−0.57	0.29	82.75%	9.99%	94.00	<0.01	H1a and H2a are supported.
	beds per doctor(BDR)	−0.28	0.04	−7.80	0.00	−0.35	−0.21					
	CMI	0.35	0.05	6.61	0.00	0.25	0.46					
	interaction	−0.39	0.04	−10.60	0.00	−0.45	−0.31					
Model 2	constant	−0.40	0.27	−1.51	0.13	−0.92	0.12	74.71%	2.18%	57.89	<0.01	H1b and H2b are support.
	beds per nurse (BNR)	−0.22	0.06	−3.65	0.00	−0.34	−0.10					
	CMI	0.23	0.06	3.83	0.00	0.11	0.35					
	interaction	−0.36	0.09	−4.11	0.00	−0.53	−0.19					
Model 3	constant	−0.34	0.26	−1.30	0.20	−0.86	0.18	74.82%	0.01%	58.23	<0.01	H1c is supported. H2c is not supported
	patients per doctor (PDR)	−0.21	0.06	−3.36	0.00	−0.33	−0.85					
	CMI	0.20	0.08	2.57	0.01	0.05	0.35					
	interaction	−0.01	0.06	−0.12	0.90	−0.14	0.12					
Model4	constant	−0.25	0.26	−0.95	0.34	−0.78	0.27	73.57%	1.13%	54.56	<0.01	H1d and H2d are not supported.
	patients per nurse (PNR)	0.14	0.10	1.41	0.16	−0.06	0.33					
	CMI	0.25	0.06	3.97	0.00	0.13	0.37					
	interaction	0.38	0.13	2.89	0.00	0.12	0.65					

LLCI, lower limit of confidence interval; ULCI, upper limit of confidence interval.

**Figure 2:**
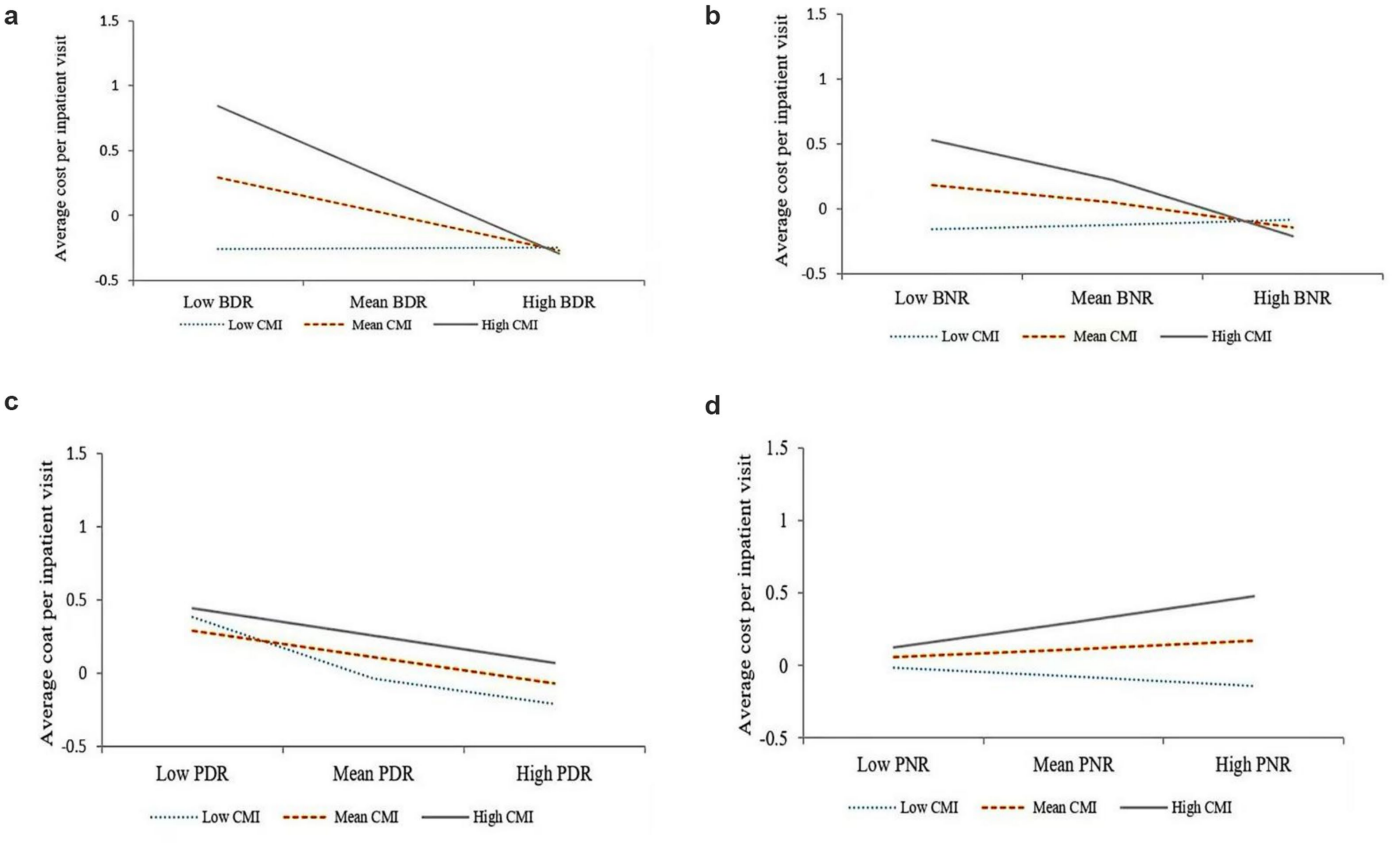
Moderating effect of CMI. **(a)** Moderating effect of CMI on the relationship between BDR and the average cost per inpatient visit. **(b)** Moderating effect of CMI on the relationship between BNR and the average cost per inpatient visit. **(c)** Moderating effect of CMI on the relationship between PDR and the average cost per inpatient visit. **(d)** Moderating effect of CMI on the relationship between NDR and the average cost per inpatient visit.

According to Aiken et al. (16), Figures 2a–d show moderating effects, where high and low levels are depicted as one standard deviation above and below the mean, respectively.

### 3.4 Robustness check

Robustness was assessed by sequentially omitting one of control variables, and the estimated moderating effect remained substantively unchanged (Table 4), confirming the stability of the results.

**Table 4:** Robustness analysis of moderating effect.

Model	Variables	B	SE	t	*p*	LLCI	ULCI	R^2^	ΔR^2^	F	*p*	Hypotheses is supported or not
Model 1	constant	−0.02	0.22	0.09	0.93	−0.42	0.46	81.04%	10.17%	93.56	<0.01	H1a and H2a are supported.
	beds per doctor(BDR)	−0.28	0.04	−7.30	0.00	−0.35	−0.20					
	CMI	0.34	0.06	6.13	0.00	0.23	0.45					
	interaction	−0.39	0.04	−10.28	0.00	−0.46	−0.31					
Model 2	constant	−0.24	0.27	−0.90	0.37	−0.77	0.29	73.07%	2.45%	59.40	<0.01	H1b and H2b are support.
	beds per nurse (BNR)	−0.22	0.06	−3.60	0.00	−0.34	−0.10					
	CMI	0.22	0.06	3.50	0.00	0.10	0.34					
	interaction	−0.38	0.09	−4.24	0.00	−0.56	−0.20					
Model 3	constant	−0.16	0.27	−0.61	0.54	−0.69	0.36	72.95%	0.01%	59.04	<0.01	H1c is supported. H2c is not supported
	patients per doctor (PDR)	−0.21	0.06	−3.37	0.00	−0.34	−0.09					
	CMI	0.17	0.08	2.16	0.03	0.01	0.33					
	interaction	−0.02	0.07	−0.31	0.76	−0.15	0.11					
Model4	constant	−0.07	0.27	−0.25	0.80	−0.61	0.47	71.51%	0.89%	54.93	<0.01	H1d and H2d are not supported.
	patients per nurse (PNR)	0.09	0.10	0.93	0.35	−0.11	0.29					
	CMI	0.23	0.06	3.49	0.00	0.10	0.35					
	interaction	0.34	0.14	2.48	0.01	0.07	0.61					

LLCI, lower limit of confidence interval; ULCI, upper limit of confidence interval.

## 4 Discussion

In the current study, the number of beds per nurse, number of beds per doctor and number of patients per doctor, which represent the level of medical staff allocation, were negatively correlated with the average cost per inpatient visit. That is, the more beds each doctor or nurse manages and the more patients each doctor manages, the lower the average cost of hospitalization per patient. The relationship between the number of patients managed per nurse and the average cost per inpatient visit was not statistically significant. This is consistent with the study of Tirgil et al. (17, 18). Rational allocation of healthcare staff by beds is not only related to the quality of care and patient safety but also to the professional satisfaction of healthcare staff and the overall operational efficiency of the healthcare organization, which in turn affects the average cost per inpatient visit (19). Christen et al. (20) demonstrated that flexibility in setting the ratio of beds to healthcare staff enables hospitals to adapt to a wide range of emergencies. However, the non-significant relationship between the number of patients managed by nurses and the average cost per visit is inconsistent with previous studies. On the one hand, this may be related to the inconsistency in the study object. For example, the study by Ross et al. (12) was based on lung cancer patients and the study by Lasater et al. (11) was based on a large sample of medical and surgical patients, whereas the present study was based on all inpatients in hospitals in Hubei Province. On the other hand, this finding may be related to the context that clinical decisions regarding treatment are typically formulated by the physician and implemented by nursing staff. Compared to the proactive role of nurses in clinical decision-making and chronic disease management in developed countries, Chinese nurses are primarily tasked with executive roles, limiting their ability to impact overall costs by optimizing the diagnostic and treatment process. Some studies (21, 22) have found that the lack of additional labor costs associated with nurses, the largest employee group in hospitals, is often offset by savings from shorter hospital stays, resulting in a limited contribution of increased nurse staffing to total costs. Further, in the Chinese healthcare system, patients are mobile and beds are fixed. A reasonable number of patients per doctor ensures that each patient receives the most appropriate treatment for their health condition, which improves treatment outcomes and reduces healthcare costs. Adjustments in the number of beds per nurse and number of beds per doctor can reflect a hospital’s ability to manage its human resources. Effective human resource management maybe improve the quality of care and medical treatment, thereby beneficial to reducing complexities and complications during hospitalization and further reducing the average cost per inpatient visit. These results provide important insight into the relationship between resource allocation and cost control and may offer a new perspective for improving resource utilization efficiency and avoiding unreasonable increases in the average cost per inpatient visit.

In addition, the results of this study indicated that the CMI was positively correlated with the average cost per inpatient visit. This is consistent with the results of previous studies (23). The higher the CMI value, the higher the severity of diseases and the greater the difficulty of diagnosis. The rapid progression of a patient’s condition requires timely and effective treatment, which consumes more medical resources and leads to higher average costs per inpatient visit. The CMI is calculated by assigning weights to different patient groups based on case complexity and resource needs. This is of use for comparing resource utilization and patient outcomes (24). A high CMI usually leads to high costs, and if a hospital has a high CMI but does not have a significant increase in the average cost per inpatient visit, this may indicate that the hospital has better cost control in dealing with complex cases.

This study examined the moderating effect of the CMI on the relationship between medical staff allocation levels and the average cost per inpatient visit. First, the CMI strengthens the effect of the number of medical staff per bed on the average cost per inpatient visit. The more beds each doctor or nurse manages, implying that the hospital spends less on staffing costs to provide more patient services, the lower the average cost per inpatient visit. Further, a greater complexity of disease implies that labor costs provide more quantity and a higher quality of services. Another possible reason for this finding is that patients with severe illnesses are quickly referred to higher-level hospitals for treatment, and thus, severely ill patients may have short hospital stays. This explanation is corroborated by Jolley et al. (25). In China, healthcare resources are mainly allocated according to the numbe r of beds. Taking the number of beds per nurse as an example, the National Nursing Career Development Plan (2021–2025) (26) stipulated that the ratio of the total number of nurses to the actual number of open beds in tertiary general hospitals should be no less than 0.85:1, and the ratio of the total number of nurses to the actual number of open beds should be no less than 0.65:1 in hospital wards. There is a similar allocation standard for the number of beds per doctor. It is noteworthy that, during the implementation of the Diagnosis-Related Groups (DRG) payment system in China, some hospitals may inflate their reported Case-Mix Index (CMI) values to secure higher reimbursement from medical insurance funds. Such upward distortion of CMI artificially amplifies the observed negative association between staffing levels and the average cost per inpatient visit, leading to the misleading impression that hospitals with more robust personnel allocation incur disproportionately higher costs. However, because the reported CMI values exceed their clinically validated counterparts, this discrepancy may generate a spurious regulatory signal, undermining the accuracy and fairness of DRG based performance assessment and resource allocation. The results of this study suggest that the allocation of healthcare professionals should be made with reference to the actual clinical severity of illnesses. Without consideration of the severity of illnesses, unreasonable average costs per inpatient visit or overworking of healthcare professionals can occur.

The CMI was not found to moderate the relationship between the number of patients per doctor and the average cost per inpatient visit. In the current study, there was a significant relationship between the interaction term (the number of patients per nurse and the CMI value) and the average cost per inpatient visit. However, H2d was not supported as there was no significant association between the number of patients per nurse and the average cost per inpatient visit. Most previous studies (11) have examined the association between the number of patients per nurse and patient outcomes such as mortality, length of stay and readmission rates. Very few scholars have examined the relationship between the number of patients per nurse and costs. One study of nurse staffing used impulse response analysis (27) and found that the number of patients per nurse had a negative effect on reimbursement costs. Future research could adopt other approaches to further explore the relationship between the number of patients per nurse and other hospital quality variables, healthcare outcome variables and healthcare cost values.

Finally, it should be noted that as indicators of staffing levels, the bed-based and patient-based staffing ratios had different impacts on the average cost per inpatient visit when moderated by the CMI. From the perspective of controlling patients’ average hospitalization costs, the current results indicate that the allocation of healthcare staff by beds may be more sensitive than the allocation of healthcare staff by patients in China. In addition, the types of diseases and criticality of patients admitted should be fully considered in the process of human resource allocation.

## 5 Strengths and limitations

The strength of this study is that it clarified the moderating role of the CMI in the relationship between healthcare resource allocation and average cost per inpatient visit. These results have implications for reasonably controlling the average cost per inpatient visit through the optimization of healthcare resource allocation with full consideration for the CMI. However, there are several limitations of this research that should be considered. First, the cross-sectional design may have introduced bias in the results. Thus, longitudinal studies are still needed to confirm the findings. Second, due to limitations with the data collection, the number of discharged patients was used as the measure of “patients” for the “number of patients per nurse” and “number of patients per doctor” measures, which may have led to large ratios. Third, this study only considered the quantity of medical and nursing staff, not the quality. Finally, the study sample comprised hospitals in Hubei Province, and although the sample size met the needs of the study, the generalizability of the results needs to be further confirmed by excluding traditional Chinese medicine hospitals, maternal and child health centers, and other institutions. In the future, the sample size should be expanded, and the sampling should be extended to other provinces in China and medical institutions of other nature to obtain more representative results. Meanwhile, given the current paucity of theoretical frameworks addressing the interrelationships among healthcare workforce allocation, Case-Mix Index (CMI), and medical expenditures, future research should prioritize the development and empirical validation of integrated theories on health resource allocation and cost determinants.

## 6 Conclusion

This study identified a relationship between medical staff allocation (the number of beds per nurse, number of beds per doctor) and the average cost per inpatient visit in general hospitals excluding traditional Chinese medicine hospitals, maternal and child health centers, and other institutions in Hubei Province. Further, a moderating effect of the CMI was found. These results provide insight into the mechanism underlying the relationship between staffing allocation and the average cost per inpatient visit in general hospitals. These findings also offer a theoretical basis for optimizing the allocation of doctors and nurses in general hospitals.

Comprehensive consideration of the CMI is beneficial for controlling growth in the average cost per inpatient visit. High CMI hospitals need to appropriately increase the allocation of doctor and nurse to ensure that critically ill patients receive adequate treatment resources, whereas excessive number of patients per doctor may be related to a decline in service quality. When controlling the growth of average hospitalization costs, CMI must be incorporated into a comprehensive consideration system. The establishment of CMI-sensitive resource allocation schemes is beneficial to improving the cost burden of patients, which has important practical value for promoting the refined management of general hospitals in China.

Simultaneously, hospitals should stratify patient care according to both their designated functional roles and their actual human resource capacity ensuring that cases of appropriate clinical complexity are managed at each level. Historically, China’s tiered healthcare system has assigned patients to institutions based primarily on disease severity and institutional function. Our research proposes that human resource allocation particularly staffing levels, skill mix, and clinical expertise must be formally integrated as a core criterion alongside functional positioning when determining case-mix appropriateness. This adjustment would help prevent the assignment of high-complexity patients (e.g., those with elevated Case-Mix Index values) to facilities lacking commensurate personnel capacity—thereby mitigating unwarranted increases in patient costs and preserving clinical efficiency.

## Data Availability

The raw data supporting the conclusions of this article will be made available by the authors, without undue reservation.
